# Mitral valve velocity time integral and passive leg raise as a measure of volume responsiveness

**DOI:** 10.1186/s13089-018-0114-3

**Published:** 2018-12-02

**Authors:** Ralphe Bou Chebl, Gilbert Abou Dagher, Jeffrey Wuhantu, Rana Bachir, Jennifer Carnell

**Affiliations:** 1 Department of Emergency Medicine, Ben Taub Hospital, Baylor College of Medicine, 1400 Taub Loop, Houston, TX USA; 2 Department of Emergency Medicine, American University of Beirut, Beirut, Lebanon

**Keywords:** Hemodialysis, LVOT, MV, VTI, Passive leg raise, Fluid responsiveness, Shock

## Abstract

**Background:**

Fluid responsiveness is an important topic for clinicians. Aggressive hydration has been shown to lead to worse outcomes. The aim of this study was to investigate the sensitivity and specificity of mitral valve (MV) velocity time integral (VTI) as a non-invasive marker of volume responsiveness.

**Methods:**

This was a prospective observational study conducted in a tertiary emergency department. End-stage renal disease patients presenting to the emergency department requiring emergent hemodialysis were enrolled. A focused echocardiogram was done on enrolled patients. Two sets of measurements were obtained before and after hemodialysis. During each scanning session, the left ventricular outflow tract and the mitral valve VTI were obtained before and after a passive leg raise maneuver.

**Results:**

54 patients were enrolled, of which, 30 (55%) were male. The mean age was 47.4 years. The mean volume of fluid removed was 3.89 ± 0.91 L. All patients had a diagnosis of hypertension, 22 (41%) patients were diabetic, 14 (26%) patients had coronary artery disease, and 19 (35%) patients had congestive heart failure. The mean change in LVOT VTI was 1.83% (95% CI 0.12–3.55) in the pre-dialysis group and 15.05% (95% CI 12.76–17.34) in the post-hemodialysis cohort. The mean change in MV VTI was 3.74% (95% CI 2.84–4.65) in the pre-dialysis cohort and 12.95% (95% CI 11.50–14.39) in the post-dialysis cohort. For patients who had < 4 L removed, the mean delta LVOT VTI post-hemodialysis was 12.64% (95% CI 9.79–15.49) and the mean delta MV VTI was 10.48% (95% CI 8.28–12.69). For patients who had > 4 L removed, the mean delta LVOT VTI was 16.84% (95% CI 13.47–20.22) and the mean MV VTI was 14.77% (95% CI 13.03–16.51). Mitral valve VTI with PLR was found to have a sensitivity of 89.18% and a specificity of 94.11% in detecting volume responsiveness.

**Conclusion:**

Mitral valve velocity time integral in conjunction with passive leg raise seem to correlate with volume responsiveness in hemodialysis patients.

## Introduction

### Background

There has been a gradual shift in the management of septic shock. Studies in the early 2000s such as the early goal-directed therapy (EGDT) advocated for aggressive fluid management guided by static measures such as the central venous pressure (CVP) [1]. Recently, however, several new studies have shown that aggressive resuscitation of septic shock patients as well as an overall positive fluid balance can be dangerous and can lead to poorer outcomes [2–4]. Moreover, relying on static measurements such as CVP to predict volume responsiveness has been put in question by multiple studies [5, 6].

This change in management has pushed researchers to look for non-invasive ways of assessing volume responsiveness. Amongst these were the inferior vena cava (IVC) ultrasound evaluation [7–10], as well as more advanced Doppler applications such as esophageal Doppler monitoring looking at changes in aortic flow time to guide fluid therapy [11, 12]. Researchers have looked at the left ventricular outflow tract (LVOT) velocity time integral (VTI) change with either a passive leg raise or a fluid bolus as a measure of volume responsiveness and found it to be specific in predicting fluid responsiveness [13, 14]. No studies, however, have looked at the role of mitral valve VTI in predicting fluid responsiveness. Acquiring an appropriate apical five-chamber view and getting an adequate window of the LVOT can be challenging. Novice emergency physicians (EP) are taught to identify the apical 4-chamber and then tilt the ultrasound upward and slightly counterclockwise to open the 5th chamber, the aorta. The inability of getting an adequate apical-5 chamber can underestimate patients’ VTI values. Given that the majority of EPs are comfortable with apical-4 views, we sought to investigate the sensitivity and specificity of mitral valve (MV) velocity time integral (VTI) as a non-invasive marker of volume responsiveness [15–17]. We evaluated the effect of the passive leg raise (PLR) maneuver on the change in MV and LVOT VTI before and after hemodialysis on patients presenting to the emergency department (ED) for emergent dialysis.

## Materials and methods

### Study design and setting

This was a prospective observational study conducted in a tertiary care emergency department. The institutional review board approved this study (Protocol # 14172). Informed consent was obtained from patients before enrolling them in the study. End-stage renal disease (ESRD) patients presenting to the emergency department requiring emergent hemodialysis were enrolled. Emergent hemodialysis was carried out based on criteria of fluid overload and hypoxia (oxygen saturation < 89%), severe acidosis (serum bicarbonate < 10 mmol/L) or severe hyperkalemia (potassium > 6 mmol/L). Patients were recruited from July to October 2017. Inclusion criteria were an age of 18 years or older and completion of a full hemodialysis session with removal of at least 2 L of fluid. Exclusion criteria included patients who were placed on non-invasive positive pressure ventilation (NIPPV), patients who were unable to tolerate the passive leg raise maneuver, patients with arrhythmias, and patients with evidence of aortic regurgitation and mitral stenosis.

### Outcomes

The primary outcome was the sensitivity and specificity of mitral valve VTI in detecting fluid responsiveness. Patients who had > 12% increase in their LVOT VTI after the passive leg raise were labeled as volume responders. Secondary outcomes included difference in the delta MV VTI pre- and post-dialysis. The delta VTI was defined as the percent change in VTI after the passive leg raise maneuver. As well as the differences in the delta LVOT VTI pre- and post-dialysis, the sensitivity, specificity, positive and negative predictive values of the MV VTI in detecting volume responsiveness. A subgroup analysis was done according to fluid removal during dialysis to look for mean delta VTI per fluid removal.

### Interventions

Study investigators were two emergency physicians with fellowship training in emergency ultrasonography and an Emergency Ultrasound fellow. The study was done at an institution with an emergency ultrasound fellowship program with special emphasis on cardiac echocardiography. All investigators completed ten ultrasound scans with the same measurements done on each scan. All three investigators reviewed all scans to standardize approach and technique before enrolling patients. All imaging measurements were performed with patients seated on a stretcher with their legs parallel to the ground and the head of the bed elevated at 45°. Before the hemodialysis session, an apical four-chamber view was obtained and a Doppler tracing of the MV blood flow was recorded with the Doppler sampling gate placed proximal to mitral valve leaflets. Following that, an apical five-chamber view was obtained and a Doppler tracing of the LVOT blood flow was recorded with the pulse wave (PW) Doppler sampling gate placed proximal to the annulus. The velocity time integral was calculated using the average of five repeated measurements over one respiratory cycle. Ultrasonographic images were obtained with a 1–5 MHz phased array transducer on a SonoSite Xporte (SonoSite, Bothell, WA).

### Measurement

Study participants’ age, sex, blood pressure, pulse rate and oxygen saturation pre- and post-dialysis as well as before and after passive leg raise (PLR) were recorded. History of co-morbidities was obtained from their medical records. Patients’ MV and LVOT VTI were measured before hemodialysis. After these measurements, a passive leg raise maneuver was performed. The head of the bed was lowered to the flat position and the patients’ legs were elevated 45° above the level of the heart. After 1 min, another set of Doppler tracing through the MV and LVOT was obtained, and the parameters were measured again. Delta VTI was defined as (VTI PLR − VTI initial)/VTI PLR. Another set of vital signs was taken after the passive leg raise maneuver. Patients were subsequently placed in the neutral position and taken to hemodialysis. After hemodialysis, a new set of vital signs, Doppler tracings and measurements were obtained before and after passive leg raise. All images were saved as still pictures, with measurements. The velocity time integral was obtained with electronic calipers in the ultrasonographic machine’s software by tracing the Doppler flow signal.

### Analysis

Based on the literature, a patient is labeled as a volume responder if their LVOT VTI increases by 12% following the PLR [14, 18–22]. Given that this is a pilot study looking at mitral valve VTI, patients with an increase in their MV VTI of ≥ 12% and < 12% were classified as responders and non-responders. Assuming a random sampling of patients, a power of 0.8 and an *α* of 0.05, a minimum sample size of 43 cases was needed. All data were analyzed using SPSS for Windows, version 17.0 (SPSS, Inc, Chicago, IL). After meeting the normality assumption (Shapiro–Wilk test), differences between values before and after hemodialysis or between supine and passive leg raise positions were calculated. Quantitative variables are presented as mean ± SD plus 95% confidence interval (95% CI) and categorical variables as frequency (percentage). Paired *t* test was performed to assess the statistical significances observed in VTI, HR, and MAP before and after hemodialysis. Independent *t* test was used to assess the statistical significance of differences observed between groups of patients with different amounts of fluid removal.

## Results

### Characteristics of study participants

**Fig. 1:**
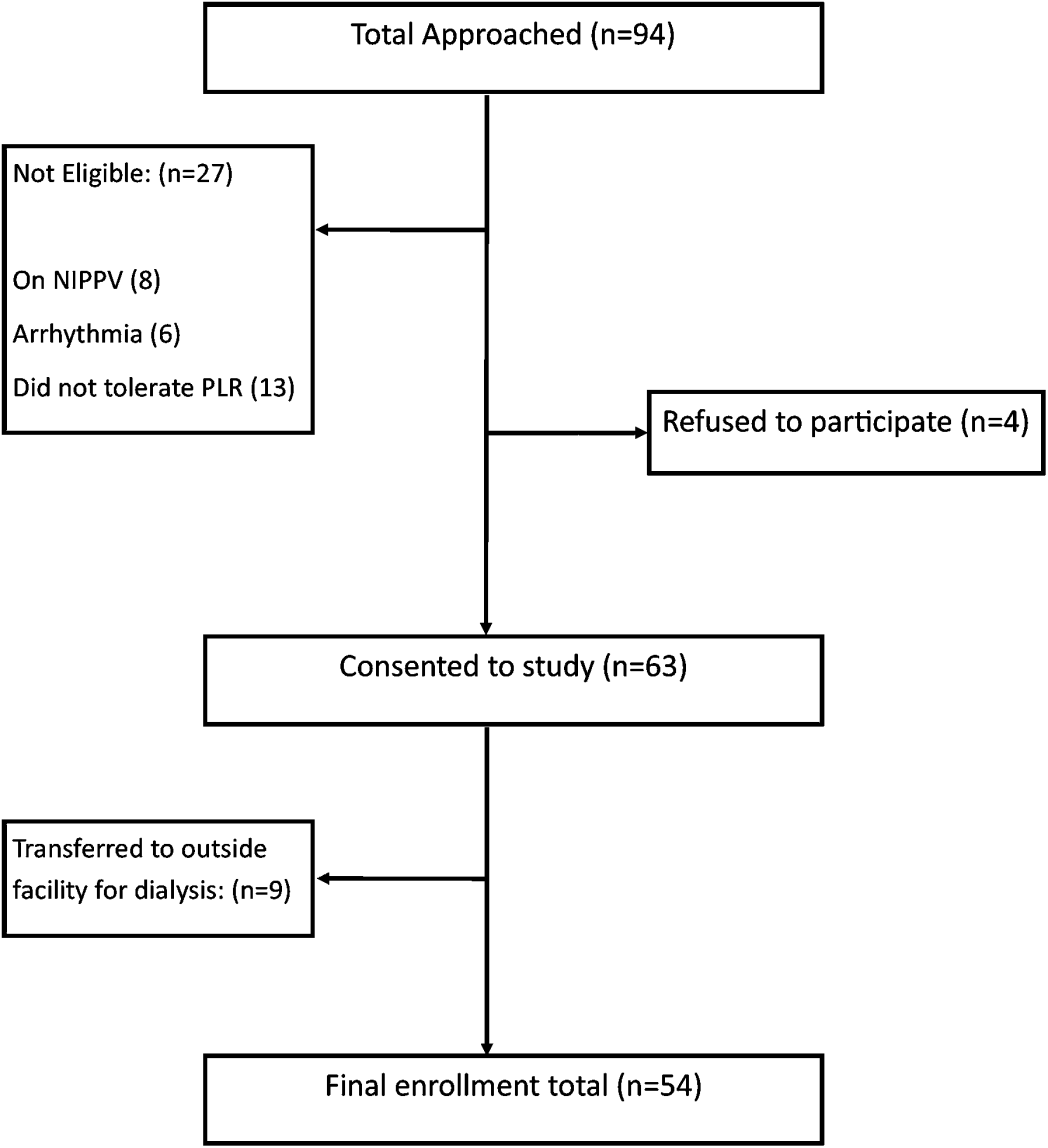
Flow diagram of patient inclusion

A total of 94 patients were approached for enrollment. 34 patients were excluded after 4 patients refused to participate, 8 were on BiPAP, 6 were found to be in atrial fibrillation, 13 patients did not tolerate the PLR maneuver, and 9 were transferred for dialysis to an outside facility (Fig. 1). A total of 54 patients were included in the study and 108 PLR challenges were done. Of all the patients included in the study, 30 (55%) were male, and the remaining 24 (45%) were female. The mean age was 47.4 years. The mean volume of fluid removed via hemodialysis was 3.89 ± 0.91 L. All patients had a diagnosis of hypertension and were on an anti-hypertensive medication. In addition to end-stage renal disease, 22 (41%) patients were diabetic, 14 (26%) patients had coronary artery disease diagnosed by cardiac catheterization. 19 (35%) patients had congestive heart failure with poor ejection fraction, which we had defined as an EF < 35%. Table 1 demonstrates patients’ demographic data.

**Table 1:** Demographics of study participants

Demographics	Mean (± SD)
Male	30 (56%)
Age (years)	47.4 ± 10.11
Hypertension	54 (100%)
Diabetes	22 (41%)
Congestive heart failure with ejection fraction < 35%	19 (35%)
CAD	14 (26%)
Asthma	1 (2%)
Days since last dialysis	7.8
Fluid removed (liters)	3.89 ± 0.91

The mean heart rate pre-dialysis was 73.43 (95% CI 70.27–76.58 bpm) and 73.91 (95% CI 71.36–76.45 bpm) post-hemodialysis. The systolic blood pressure before dialysis was 157.78 (95% CI 151.45–164.11 mmHg) and 165.69 (95% CI 159.91–171.46) after. The mean diastolic blood pressure before dialysis was 85.19 (95% CI 80.60–89.77) and 83.74 (95% CI 77.23–90.25) after. The complete list of vitals is provided in Table 2.

**Table 2:** Vital signs of study participants

Vitals	Pre-dialysis (*N* = 54)		Post-dialysis (*N* = 54)	
	Mean	95% CI	Mean	95% CI
HR	73.43	70.27–76.58	73.91	71.36–76.45
SBP	157.78	151.45–164.11	165.69	159.91–171.46
DBP	85.19	80.60–89.77	83.74	77.23–90.25
O_2_ saturation	96.35	95.32–97.38	97.98	97.29–98.68
HR PLR	73.94	70.72–77.17	74.67	72.19–77.14
SBP PLR	159.43	153.03–165.82	168.43	162.79–174.06
DBP PLR	85.74	80.83–90.65	87.26	82.56–91.96
O_2_ saturation PLR	96.17	95.14–97.20	97.87	97.11–98.63

### Main results

A total of 32 patients were labeled as volume responders with a delta LVOT VTI > 12% in the post-dialysis group. Mitral valve VTI in conjunction with a passive leg raise was found to have a sensitivity of 89.18%, a specificity of 94.11%, a positive predictive value of 97.05% and a negative predictive value of 80% in predicting volume responsiveness.

In the pre-dialysis cohort, the mean LVOT VTI was 28.05 cm (95% CI 26.55–29.55). Following the PLR maneuver, the mean LVOT VTI was 28.52 cm (95% CI 26.98–30.07). The mean MV VTI was 30.08 cm (95% CI 28.44–31.71) and after PLR, it was 31.24 cm (95% CI 29.58–32.90). In the post-hemodialysis cohort the mean LVOT VTI was 30.31 cm (95% CI 28.92–31.69). Following the PLR maneuver, the mean LVOT VTI was 34.91 cm (95% CI 33.11–36.72). The mean MV VTI was 30.11 cm (95% CI 28.47–31.67) and it was 34.70 cm (95% CI 32.56–36.48) following the PLR maneuver. In the pre-dialysis group, the delta LVOT VTI was found to be 1.83% (95% CI 0.12–3.55) and the delta MV VTI was 3.74% (95% CI 2.84–4.65), while in the post-dialysis group, the delta LVOT VTI was 15.19% (95% CI 12.76–17.34) and the delta MV VTI was found to be 12.95% (95% CI 11.50–14.39). Patients were divided according to fluid removal. The mean increase in LVOT VTI was 12.64% (95% CI 9.79–15.49). For patients who had less than 4 L removed, the MV VTI increased by 10.48% (95% CI 8.28–12.69). For patients who had more than 4 L removed, the mean increase in LVOT VTI was 16.84% (95% CI 13.47–20.22) and 14.77% (95% CI 13.03–16.51) for the MV VTI. These results are summarized in Table 3.

**Table 3:** Mitral valve and left ventricular outflow tract velocity time integral changes before and after dialysis

	Pre-dialysis (*N* = 54)		Post-dialysis (*N* = 54)		*p* value
	Mean	95% CI	Mean	95% CI	
MV VTI	30.08	28.44–31.71	30.11	28.47–31.67	0.907
MV VTI PLR	31.24	29.58–32.90	34.70	32.56–36.48	0.012
DELTA MV VTI	3.74	2.84–4.65	12.95	11.50–14.39	< 0.001
LVOT VTI	28.05	26.55–29.55	30.31	28.92–31.69	0.029
LVOT VTI PLR	28.52	26.98–30.07	34.91	33.11–36.72	< 0.001
DELTA LVOT VTI	1.83	0.12–3.55	15.19	12.76–17.34	< 0.001

## Discussion

The results of this study have shown that in hemodialysis patients, non-invasive bedside echocardiography coupled with a passive leg raise is helpful in predicting volume responsiveness. In the pre-dialysis cohort of patients, the passive leg raise maneuver did not result in any VTI changes, whether at the level of the LVOT or at the mitral valve, whereas, after the fluid was removed, the delta LVOT VTI increased by 15% with PLR and the delta MV VTI increased by 12.95%. Our results also showed that the delta VTI increase was volume dependent as patients who had greater than 4 L removed had the greatest increase in delta VTI.

Fluid responsiveness and fluid management in shock are important topics for intensivists and emergency physicians. Aggressive hydration in the ED became the mainstay of septic shock therapy after the EGDT trial [1]. The Rivers protocol was recently criticized and challenged by several studies that showed that aggressive fluid therapy in sepsis can be dangerous and can lead to increased mortality [2–4, 23, 24]. Furthermore, the use of static measures such as the central venous pressure (CVP) in predicting fluid responsiveness has been put to question [5, 6]. Fluid responsiveness is an even more pivotal issue in patients who are chronically fluid overloaded such as ESRD patients. When these patients present hypotensive due to sepsis, it is difficult for the treating physician to gauge their intravascular volume status through the use of physical exam findings and through static measures (mean arterial pressure, CVP) [25, 26]. This has prompted researchers to look for new methods of assessment of volume responsive. One of the first methods looked at aortic flow changes using an esophageal Doppler. While it showed a direct correlation with intravascular volume changes, this technique remained highly invasive, as the patient needed to be intubated or sedated [12]. More recently, there has been a gradual shift towards non-invasive dynamic ways of assessing volume responsiveness. Physicians looked at the inferior vena cava (IVC) collapsibility as a surrogate marker of volume responsiveness. While early studies showed great correlation between IVC collapsibility and patient’s volume status, the majority of studies did not show a great correlation with fluid responsiveness in spontaneously breathing patients [8–10, 27–29]. More recently, a group lead by Lamia et al. [13] looked at the role of non-invasive echocardiography as a measure of volume responsiveness in ICU, and they showed that a 12.5% increase in VTI was 77% sensitive and 100% specific for detection of a > 15% in cardiac output following volume expansion. These similar findings were reproduced by Maizel et al. [14] who showed that in spontaneously breathing patients presenting in shock, they showed that a 12% increase in stroke volume after passive leg raise was 69% sensitive and 89% specific for response to 500 mL of crystalloid administration. To the best of our knowledge, our study is the first study looking at the role of non-invasive cardiac echocardiography in the emergency department for the evaluation of volume responsiveness. A study by Dinh et al. in 2012 showed that emergency physicians can accurately measure LVOT VTI and cardiac output [30]. Our study remains the only study that looked at the role of MV VTI as a predictor of volume responsiveness. MV VTI was found to be highly specific for fluid responsiveness as well as having a high positive predictive value for detecting a volume-responsive state. This technique can be an alternative for physicians to evaluate volume responsiveness in cases where patients’ body habitus prevents them from getting an adequate apical five-chamber view. It is important to note that there were four patients that had a MV VTI < 12% while having an LVOT VTI > 12%. Possible explanations for the discrepancy could be that the patients had diastolic dysfunction or mitral regurgitation, two conditions that could affect mitral valve VTI. We did not, however, check for diastolic dysfunction on our patients. Furthermore, one patient had a MV > 12% and an LVOT VTI < 12%. This particular patient had an aortic valve replacement and his low LVOT VTI could be due to the metallic valve.

In contrast to the prior VTI studies, we removed fluid via hemodialysis and we looked at the effect of the passive leg raise once these patients had this fluid taken off. The rationale was that in the pre-dialysis cohort, patients would be hypervolemic and their overstretched heart will not respond to the PLR maneuver and the preload bolus, in contrast to the post-hemodialysis cohort, which would represent a fluid responsive state and would allow the heart to respond to the preload challenge and increase its stroke volume. The passive leg raise maneuver by definition is a reversible auto-bolus of about 200–400 mL. It has been studied in both ventilated and spontaneously breathing patients, and several studies have shown that an increase in stroke volume of 15% following a PLR maneuver had a specificity of 93% in detecting volume responsiveness after receiving a 500-cc fluid bolus [21, 22, 31]. Furthermore, we chose to conduct this study on end-stage renal disease because this subset of patients represents a challenge while being evaluated for fluid responsiveness, as these patients are chronically fluid overloaded and still can present with hypotension and it is often unclear whether these patients require more fluid or vasopressor therapy.

It is interesting to note that there were no differences in vital signs before and after hemodialysis except for systolic blood pressure, which we noted to be higher by 8 mmHg in the post-dialysis cohort. This increase in systolic blood pressure after dialysis was described by Inrig et al. [32] who explained that intradialytic hypertension is multifactorial, and its causes include subclinical volume overload, sympathetic overactivity, activation of the renin angiotensin system, endothelial cell dysfunction, and specific dialytic techniques. It is important to note that this increase in blood pressure is only temporary. In the majority of patients, there is a drop in afterload post-dialysis coupled with an increase in their cardiac output [33]. This could explain why our VTI values increased in the post-hemodialysis cohort. However, it is more important to note that there were no changes in vital signs after the passive leg raise signs which further strengthens the idea that vital sign changes are not adequate enough for assessing volume responsiveness. This has been shown several times in the literature and in previous studies on bedside echocardiography and further strengthens the argument that VTI changes are more sensitive during volume changes than vital sign changes [12, 13, 22].

The greatest increase in the delta VTI for both the mitral valve and the LVOT in our population was seen in patients who had more than 4 L of fluid removed. This can probably be due to several reasons; first, the interval between dialysis sessions was longer in our patients (7.8 days) than regular hemodialysis patients. Our patients receive hemodialysis on a compassionate or emergent basis based on criteria of fluid overload and hypoxia, severe acidosis (serum bicarbonate < 10 mmol/L0 or severe hyperkalemia (Potassium > 6 mmol/L). As such, they usually go more than the standard 3 days without dialysis and might be more fluid overloaded than the scheduled hemodialysis patient. Another possible explanation is that hemodialysis is done over a period of 3–4 h which could allow the system time to slowly adapt to the volume loss.

The present study is an observational study performed on a specific group of ESRD patients with volume overload. Therefore, its findings should be interpreted cautiously as they are not applicable to the general ED population. Our results should be compared with the results of future studies on other populations with hypervolemia or hypovolemia. Furthermore, our study is limited by its small sample size; however, this sample size was calculated based on the existing literature on volume responsiveness and velocity time integral. Moreover, considering ESRD patients after hemodialysis as euvolemic may not be accurate. Our study is also limited by the lack of lung ultrasonography. Although our aim was to investigate the value of mitral valve VTI, several studies have looked at the role of lung ultrasound in ESRD patients as a surrogate for volume status [34–36]. Future studies need to combine cardiac echo with lung ultrasonography in an effort to best understand volume responsiveness. Finally, the ultrasound examination was performed by physicians with extensive training in bedside ultrasound and therefore cannot be generalized to all emergency physicians.

## Conclusion

Volume status evaluation of hemodialysis patients can be tricky as reliance on vital signs and the physical exam are not very accurate. Bedside echocardiography in conjunction with a passive leg raise is a relatively simple, non-invasive method that could help in evaluating for volume responsiveness and, therefore, is a valuable tool for intensivists. Further large studies, however, are needed to corroborate our findings.

## Abbreviations

MV: mitral valve
LVOT: left ventricular outflow tract
VTI: velocity time integral
PLR: passive leg raise
ESRD: end-stage renal disease
NIPPV: non-invasive positive pressure ventilation
HR: heart rate
MAP: mean arterial pressure
BP: blood pressure
CVP: central venous pressure
IVC: inferior vena cava
EGDT: early goal-directed therapy
